# Evaluating factors associated with awareness and attitude towards pre-exposure prophylaxis among cisgender sexually active women in Ghana

**DOI:** 10.1186/s12889-025-25303-6

**Published:** 2025-11-21

**Authors:** Michael Deynu, Jerry John Ouner

**Affiliations:** Department of Family Health Care Nursing, School of Nursing, University of California San Francisco, San Francisco, USA

**Keywords:** Pre-exposure prophylaxis, Human immunodeficiency virus, HIV prevention, Cisgender women, Demographic and health survey, Ghana

## Abstract

**Background:**

HIV remains a public health issue globally and women continue to be disproportionately impacted. Pre-exposure prophylaxis (PrEP) is one critical intervention aimed at HIV prevention. However, awareness on PrEP remains suboptimal in most resource poor countries. Understanding the factors associated with PrEP awareness among sexually active women is an important part of the HIV prevention strategy. Therefore, we examined factors associated with awareness and attitude towards PrEP among cisgender sexually active women in Ghana.

**Methods:**

This was a secondary analysis of the 2022 Ghana demographic and health survey (DHS) data involving a total of 3981 sexually active women aged 15–49 years. Percent and multiple logistic regression models were fitted to estimate prevalence and factors associated with PrEP awareness. All analyses were conducted using the “svy” command in STATA, version 18. Results were reported as adjusted odds ratio (aOR) and at 95% confidence interval (CI).

**Results:**

Awareness on PrEP was low (20.0%). Sexually active women married/living with their partner were associated with 0.68 times the odds of PrEP awareness (0.47, 0.98). Women living in rural areas were associated with 0.60 times the odds of being aware of PrEP (0.47, 0.77). Women in the high-income bracket were associated with 1.50 higher odds of being aware of PrEP (1.17,1.93). Access to newspaper (1.84 [1.06, 3.21]), radio (1.88 [1.43, 2.47]) and television sets (1.55 [1.14, 2.09]) were associated with greater odds of PrEP awareness. Women who had heard about other STIs (1.64 [1.20, 2.24]), visited health facility (1.47 [1.18, 1.84]), visited by healthcare worker (1.50 [1.12, 2.02]), were aware of anti-retroviral therapy (ART) to treat HIV (3.86 [2.69, 5.54]), and had heard of HIV test kits (5.09 [3.50, 7.39]) were associated with greater odds of being aware of PrEP. Women with higher education were associated with greater odds of being aware about PrEP (3.34 [2.25, 4.95]).

**Conclusions:**

PrEP awareness among sexually active women in Ghana is low. Our findings can inform implementation of awareness campaigns on PrEP and increase its use and adherence among key populations.

## Background

Human Immune-deficiency Virus (HIV) remains a global public health issue [1], with an estimated 39.9 million people living with HIV, of whom 65% are in the World Health Organization (WHO) African Region [2]. In 2023 alone, an estimated 1.3 million people became infected with HIV while nearly 630 000 reportedly died from HIV-related causes [2, 3]. Among people living with HIV, women and young girls accounted for 53% as well as 44% for all new HIV infections [3]. In Ghana, nearly 210 000 women aged 15 years and over were living with HIV in 2023; approximately twice the burden in men (110 000) [4]. Similarly, current estimates showed a 2.0% HIV prevalence among women compared to 1.0% among men in Ghana; with a similar trend among young women (0.7%) compared to young men (0.4%) [4]. Generally, women are disproportionately impacted by the HIV epidemic compared to men in sub-Saharan Africa, SSA [5, 6].

Pre-Exposure Prophylaxis (PrEP) is one efficacious intervention aimed at preventing HIV [7–9]. Further, current clinical trials have also demonstrated the effectiveness of twice-yearly injectable PrEP, Lenacapavir in preventing HIV among cisgender women [10]. The scale up and adoption of HIV prevention and PrEP services is an important strategy for the global target of ending the HIV epidemic by 2030 [11]. Despite its effectiveness, awareness on PrEP and its use has rather been low across SSA among key populations including women [12–18]. Fear of side effects, cost of PrEP, stigma, lack of friendly services, and long waiting times are some common barriers to PrEP uptake and adherence [19–23]. Further, risk perception, HIV testing, high income, having a partner who is positive, residence, and educational attainment were also reported to influence PrEP uptake and adherence [24–27]. In Ghana however, limited studies have examined PrEP and its use among the population [28–33]. Across these studies, majority were qualitative studies with large proportion focused on sexual minority men (SMM), providers and young men.

In the current literature, and to our knowledge, no study has been identified to have examined awareness and attitude towards PrEP among sexually active cisgender women in Ghana particularly using large population-level survey data. Given that women in Ghana are disproportionately impacted by the HIV epidemic compared to men [4], it is important to examine awareness and attitude towards PrEP, an important HIV prevention strategy among this population. Findings from this study could help inform development and implementation of targeted interventions aimed at increasing PrEP awareness, use and adherence among sexually active women in Ghana. Our analysis, therefore, assessed awareness and attitude towards PrEP among sexually active cisgender women in Ghana.

## Methods

### Study design and setting

This is a secondary cross-sectional analysis of the 2022 Ghana Demographic and Health Survey (DHS) data, a national and representative data collected across the 16 administrative regions of Ghana [34]. Ghana is a country located on the West African coast and borders Togo to the East, Ivory Coast to the West, Burkina Faso to the North and the Gulf of Guinea to the South.

### Study participants and data sources

We analyzed a sample of 3981 sexually active (last 4 weeks prior to the survey) cisgender women aged 15–49 years. We extracted data for this sample from the women’s questionnaire of the 2022 GDHS survey which collected information on maternal and child health, fertility, contraceptive use, neonatal, infant, child and mortalities, HIV/AIDS awareness, maternal mortality, gender, nutrition and sexually transmitted infections (STIs) amongst others from reproductive women aged 15–49 years from 17,933 households [34]. The data collection spanned October 2022-January 2023 [34]. The Ghana DHS data is publicly available.

### Sampling procedures

The 2021 Ghana Population and Housing Census (PHC) by the Ghana Statistical Service (GSS) provided the sampling frame for the 2022 GDHS. The 2022 GDHS data was collected through a multi-stage sampling approach ensuring that nationally representative data was generated across urban and rural areas in each of the 16 administrative regions of Ghana. Samples were selected in each sampling stratum through a two-stage selection. In the first stage, 618 enumeration areas (EAs) were selected with a probability proportional to size selection procedure after which household listings were conducted. The list of households served as the sampling frame for the second stage of sample selection. In the second stage of sample selection, 30 households were selected in each urban and rural cluster through equal probability systematic sampling based on the updated household listing for the final interviews. The detailed methodology of the survey design, sampling and data collection methods in the original 2022 GDHS survey is described elsewhere [34].

### Measures

The main outcome was awareness and attitude towards pre-exposure prophylaxis (PrEP) among sexually active women in Ghana. Data on this question was collected in the DHS survey by asking participants the following questions “ Have you heard of PrEP, a medicine taken daily that can prevent a person from getting HIV? and “Do you approve of people who take a pill every day to prevent getting HIV? The outcome variable was coded as binary outcome (Not aware and have negative attitude towards PrEP/Aware and have positive attitudes towards PrEP). These was merged to create a composite variable known as “awareness and attitude towards PrEP” in the Ghana 2022 DHS dataset.

### Potential correlates

We included socio-behavioral, and demographic characteristics based on the framework of the Information, Motivation and Behavioral Skills Model (IMB) [35], their relevance in prior research [12, 15, 36–41] and their availability in the 2022 GDHS dataset. These included age (15–24 years, 25–34 years, 35–44 years and 45 + years), marital status (never married, married/living with partner, widowed/divorced), place of residence (urban, rural), region of residence (Western, Central, Greater Accra, Volta, Eastern, Ashanti, Western North, Ahafo, Bono, Bono East, Oti, Northen, Savannah, North East, Upper East, Upper West regions), highest educational level (no education, primary, secondary, higher education), wealth index (low, middle and high), ethnicity (Akan, Ga/Dangme, Ewe, Guan, Mole-Dagbani, Grusi, Gurma, Mande & Others).

Further, we included religion (Catholics/Presbyterian, Pentecostals, Islam and others), currently working (yes, no), frequency of reading newspaper/magazine (not at all, less than once a week, at least once a week), frequency of listening to radio (not at all, less than once a week, at least once a week), frequency of watching TV (not at all, less than once a week, at least once a week), had any sexually transmitted infections (STIs) in the last 12 months (yes, no), had any genital sore/ulcer in the last 12 months (yes, no), had any genital discharge in the last 12 months (yes, no), number of sexual partners excluding spouse in the last 12 months (none, one (1), more than two (2)), condom use during last sex with most recent partner (yes, no), heard about other STIs (yes, no), visited health facility in the last 12 months (yes, no), visited by healthcare worker in the last 12 months (yes, no), heard of ART to treat HIV (yes, no), and Knowledge and use of HIV test kits (never heard of hiv test kits, has heard of hiv test kits, knows hiv test kits but never tested with them).

### Data analysis

We extracted data from the 2022 GDHS dataset. We conducted data cleaning and missingness assessment, after which we conducted univariate analysis to describe participant characteristics using frequencies and percentages. Pearson’s chi square statistics were used to determine the association between the outcome and exposures. We then conducted a crude logistic regression analysis to examine the association between our outcome and predictors. We then fitted multiple logistic regression models with our outcome and exposures while we controlled for age, religion, ethnicity and respondent working status as potential covariates to examine the independent effect of the exposures on awareness and attitude towards PrEP among cisgender women in Ghana.

This approach was adopted to avoid reporting effect estimates for both primary exposures and other covariates from the same statistical model which may be misleading and resulting in incorrect interpretations [42, 43]. Level of significance was set at *p* < 0.05. Potential correlates were selected into the multiple regression model based on *p* < 0.05, theoretical relevance and clinical knowledge. Results of multiple regression analysis were reported as adjusted odds ratio (aOR) and at 95% confidence interval (95% CI). We evaluated potential effect modification by income level, educational level, visiting healthcare facility in the last 12 months and healthcare worker visitation by the inclusion of interaction terms with the main exposures. All analyses were conducted using STATA 18.0 survey estimation function (“svy” command) to account for survey design and to obtain nationally representative estimates [44–46].

## Results

### Demographic characteristics of study participants

In all, 3981 sexually active cisgender women were included in the study (Table 1). In our sample, 41% were aged 25–34 years while 15.24% were aged 15–24 years. The majority were married/living with partner (85.74%). Further, 58.93% were living in urban areas. More than half (58.62%) reported having attained secondary education. Nearly 53% were in the high wealth index. Additionally, 86.51% reported they were employed while 90% indicated that they had not read newspaper or magazine.

**Table 1:** Participant demographics and bivariate distributions of awareness and attitudes towards PrEP, (N=3981)

**Characteristics **	**Frequency (N) %**	**Awareness and attitude towards PrEP**		
		**No (N=3164)**	**Yes (N=817)**	**P-value**
Participant age (years)				0.01
15-24	664 (15.24)	562(16.22)	102(11.34)	
25-34	1681(40.72)	1285(39.20)	396(46.71)	
35-44	1339(35.56)	1075(35.92)	264(34.16)	
45+	297(8.48)	242(8.66)	55(7.79)	
Marital Status				0.27
Never in union	350(9.21)	267(8.85)	83(10.67)	
Married/Living with partner	3461(85.74)	2752(85.85)	709(85.33)	
Widowed/Divorced	170(5.04)	145(5.30)	25(4.00)	
Place of residence				0.00
Urban	2110(58.93)	1614(56.47)	496(68.68)	
Rural	1871(41.07)	1550(43.53)	321(31.32)	
Region of residence				0.00
Western	271(7.50)	239(8.22)	32(4.67)	
Central	269(10.75)	198(9.91)	71(14.10)	
Greater Accra	319(17.36)	286(17.89)	51(15.25)	
Volta	247(4.97)	180(4.51)	67(6.79)	
Eastern	313(10.36)	266(10.90)	47(8.22)	
Ashanti	353(20.96)	288(21.57)	65(18.55)	
Western North	247(3.01)	203(3.07)	44(2.80)	
Ahafo	220(1.91)	162(1.72)	58(2.70)	
Bono	238(3.68)	201(3.91)	37(2.78)	
Bono East	225(3.69)	184(3.75)	41(3.48)	
Oti	242(2.52)	183(2.47)	59(2.74)	
Northen	205(4.67)	120(3.48)	85(9.37)	
Savannah	141(0.99)	127(1.13)	14(0.47)	
North East	184(1.38)	157(1.45)	27(1.13)	
Upper East	282(4.03)	211(3.82)	71(4.86)	
Upper West	225(2.17)	177(2.20)	48(2.09)	
Highest Educational level				0.00
No education	720(13.42)	598(14.03)	122(11.02)	
Primary	624(14.74)	528(15.86)	96(10.29)	
Secondary	2136(58.62)	1738(59.86)	398(53.71)	
Higher	501(13.22)	300(10.25)	201(24.98)	
Wealth Index				0.00
Low	1433(25.98)	1177(27.08)	256(21.66)	
Middle	860(21.56)	707(22.68)	153(17.12)	
High	1688(52.46)	1280(50.24)	408(61.23)	
Ethnicity				0.00
Akan	1567(48.78)	1244(48.11)	323(51.40)	
Ga/Dangme	217(8.57)	191(9.52)	26(4.85)	
Ewe	477(11.94)	373(11.69)	104(12.91)	
Guan	155(2.57)	131(2.79)	24(1.69)	
Mole-Dagbani	899(15.64)	663(14.44)	236(20.38)	
Grusi	216(3.44)	189(3.79)	27(2.11)	
Gurma	330(5.38)	271(5.66)	59(4.29)	
Mande & Others	120(3.67)	102(4.00)	18(2.37)	
Religion				0.44
Catholics/Presbyterians	858(21.03)	666(21.04)	192(20.98)	
Pentecostals	1526(43.17)	1230(43.13)	296(43.36)	
Islam	865(16.67)	670(16.10)	195(18.90)	
Others	732(19.13)	598(19.73)	134(16.76)	
Respondent current working				0.09
No	588(13.49)	473(14.04)	115(11.34)	
Yes	3393(86.51)	2691(85.96)	702(88.66)	
Frequency of reading Newspaper/Magazine				0.00
Not at all	3618(89.74)	2931(91.52)	687(82.71)	
Less than once a week	253(7.33)	155(5.91)	98(12.95)	
At least once a week	110(2.93)	78(2.57)	32(4.34)	
Frequency of listening to radio				0.00
Not at all	1252(27.71)	1061(29.63)	191(20.12)	
Less than once a week	960(24.79)	768(25.29)	192(22.80)	
At least once a week	1769(47.5)	1335(45.08)	434(57.08)	
Frequency of watching TV				0.00
Not at all	924(17.53)	779(818.66)	145(13.04)	
Less than once a week	565(13.95)	452(14.23)	113(12.81)	
At least once a week	2492(68.53)	1933(67.11)	559(74.15)	
Had any STI’s in the last 12 months				0.93
No	3661(92.59)	2912(92.61)	749(92.50)	
Yes	320(7.41)	252(7.39)	68(7.50)	
Had genital sore/ulcer in the last 12 months				0.57
No	3585(90.86)	2842(90.71)	743(91.45)	
Yes	396(9.14)	322(9.29)	74(8.55)	
Had genital discharge in the last 12 months				0.20
No	3098(77.86)	2445(77.30)	653(80.07)	
Yes	883(22.14)	719(22.70)	164(19.93)	
Number of sex partners excluding spouse in last 12 months				0.31
None	3366(83.22)	2667(82.92)	699(84.41)	
1	559(15.38)	449(15.51)	110(14.84)	
2+	56(1.40)	48(1.57)	8(0.75)	
Condom used during last sex with most recent partner				0.08
No	3847(96.88)	3070(97.17)	777(95.71)	
Yes	134(3.12)	94(2.83)	40(4.29)	
Heard about other STI’s				0.00
No	761(16.24)	660(17.45)	101(11.43)	
Yes	3220(83.76)	2504(82.55)	716(88.57)	
Visited health facility last 12 months				0.00
No	1426(37.95)	1177(39.75)	249(30.81)	
Yes	2555(62.05)	1987(60.25)	568(69.19)	
Visited by fieldworker in last 12 months				0.00
No	3249(81.19)	2639(82.63)	610(75.46)	
Yes	732(18.81)	525(17.37)	207(24.54)	
Heard of ARTs to treat HIV				0.00
No	965(22.81)	904(26.50)	61(8.20)	
Yes	3016(77.19)	2260(73.50)	756(91.80)	
Knowledge and use of HIV test kits				0.00
Never heard of HIV test kits	3077(77.99)	2607(82.63)	470(59.61)	
Has heard of HIV test kits	181(3.77)	90(2.35)	91(9.44)	
Knows HIV test kits but never tested with them	723(18.24)	467(15.02)	256(30.95)	
Awareness and attitude towards PrEP				
Not aware and have negative attitude towards PrEP	3164(79.83)			
Aware and have positive attitudes towards PrEP	817(20.17)			

Also, 68.53% indicated having watched TV at least once a week. Majority 83.22% reported they had no other sexual partner aside their spouse while 96.88% indicated no condom use during their last sexual encounter with their most recent partner. Also, 83.76% indicated having heard about STIs while 81.19% reported they had not been visited by a health field worker. Further, 77.19% of women reported having heard of anti-retroviral therapy (ART) to treat HIV. Although majority were aware of ART to treat HIV, 77.99% reported that they had not heard of HIV test kits whilst 18.24% indicated that they were aware of HIV self-test kits but never used them.

### Bivariate distribution of awareness and attitude towards PrEP by socio-demographic characteristics, *N* = 3981

Of our total sample, 46.71% of the participants aged 25–34 years while 11.34% aged 15–24 years indicated being aware of PrEP, (*p* = 0.01) (Table 1). Further, 68.68% of sexually active women living in urban areas indicated being aware of PrEP compared to 31.32% living in rural areas, (*p* < 0.001). More than half (53.71%) of women with secondary level education reported being aware of PrEP, (*p* < 0.001). The majority, 61.23% of women in the high wealth index indicated being aware of PrEP compared to 21.66% in the low wealth index, (*p* < 0.001). Further, 12.95% and 4.34% of women who had read a newspaper less than once a week and at least once a week reported being aware of PrEP, (*p* < 0.001), respectively.

Similarly, 57.08% of our sample who had listened to radio at least once a week were aware of PrEP whilst 74.15% of women who reported watching Television (TV) at least once a week indicated being aware of PrEP, (*p* < 0.001). Further, 88.57% of our sample who reported having heard about other STIs indicated that they were aware of PrEP, (*p* < 0.001). For participants who reported visiting the health care facility in the last 12 months, 69.19% indicated that they were aware of PrEP, (*p* < 0.001). Similarly, 24.54% of women who indicated they were visited by health fieldworker reported PrEP awareness, (*p* < 0.001).

Also, 91.80% of sexually active cisgender women with knowledge on ARTs to treat HIV were aware of PrEP, (*p* < 0.001). Additionally, 9.44% of women who had heard about HIV test kits were aware of Pre-Exposure Prophylaxis (PrEP), (*p* < 0.001).

### Correlates of awareness and attitude towards PrEP among sexually active cisgender women in Ghana

Our multiple binary logistic regression showed that marital status, place of residence, region of residence, educational level, wealth index, access to media, awareness about other STIs, visiting health facility, visited by fieldworker, having heard about ARTs to treat HIV and awareness and use of HIV test kits were associated with PrEP awareness among sexually active cisgender women (Table 2). Sexually active cisgender women who were married/living with their partner compared to those who are not were associated with 0.68 lower odds of being aware of PrEP (0.68 [0.47–0.98], *p* = 0.04). Also, women living in rural areas compared to those in urban areas were associated with 0.60 lower odds of PrEP awareness (0.60 [0.47–0.77], *p* < 0.001). Sexually active cisgender women living in the Volta region of Ghana compared to those in the Western region were associated with 3.72 times greater odds of being aware of PrEP (3.72 [1.86–7.49], *p* < 0.001).

**Table 2:** Correlates of awareness and attitude towards PrEP among sexually active cisgender women in Ghana

**Characteristics**	**Awareness and attitude towards PrEP**					
	**COR**	**95% CI**	***P*** **-value**	**aOR**	**95% CI**	***P*** **-value**
Marital Status						
Never in union	ref	ref		ref	ref	
Married/Living with partner	0.82	0.58-1.17	0.28	**0.68**	**0.47-0.98**	**0.04**
Widowed/Divorced	0.62	0.35-1.13	0.12	0.55	0.30-1.00	0.05
Place of residence						
Urban	ref	ref		ref	ref	
Rural	0.59	0.46-0.76	<0.001	**0.60**	**0.47-0.77**	**<0.001**
Region of residence						
Western	ref	ref		ref	ref	
Central	2.50	1.41-4.46	0.00	**2.74**	**1.53-4.91**	**0.001**
Greater Accra	1.50	0.81-2.76	0.19	**2.05**	**1.08-3.87**	**0.03**
Volta	2.65	1.48-4.74	0.00	**3.72**	**1.86-7.49**	**<0.001**
Eastern	1.33	0.75-2.35	0.33	1.68	0.93-3.03	0.08
Ashanti	1.51	0.82-2.79	0.19	1.65	0.88-3.08	0.12
Western North	1.60	0.87-2.95	0.13	1.74	0.95-3.19	0.08
Ahafo	2.76	1.53-4.50	0.00	**3.25**	**1.82-5.83**	**<0.001**
Bono	1.24	0.70-2.21	0.46	1.33	0.74-2.39	0.34
Bono East	1.63	0.85-3.13	0.14	**2.17**	**1.10-4.26**	**0.03**
Oti	1.95	1.08-3.53	0.03	**3.86**	**1.96-7.59**	**<0.001**
Northen	4.73	2.26-9.88	0.00	**6.07**	**2.94-12.55**	**<0.001**
Savannah	0.74	0.39-1.40	0.35	1.21	0.58-2.50	0.61
North East	1.37	0.59-3.16	0.46	1.75	0.79-3.88	0.17
Upper East	2.24	1.21-4.16	0.01	**3.26**	**1.63-6.49**	**0.001**
Upper West	1.67	0.91-3.05	0.09	**2.21**	**1.18-4.16**	**0.01**
Highest Educational level						
No education	ref	ref		ref	ref	
Primary	0.83	0.55-1.24	0.35	0.93	0.63-1.39	0.73
Secondary	1.14	0.80-1.63	0.47	1.31	0.91-1.89	0.15
Higher	3.09	2.11-4.54	<0.001	**3.34**	**2.25-4.95**	**<0.001**
Wealth Index						
Low	ref	ref		ref	ref	
Middle	0.94	0.70-1.27	0.70	0.95	0.70-1.27	0.71
High	1.52	1.18-1.97	0.00	**1.50**	**1.17-1.93**	**0.00**
Frequency of reading Newspaper/Magazine						
Not at all	ref	ref		ref	ref	
Less than once a week	2.42	1.65-3.57	<0.001	**2.63**	**1.75-3.95**	**<0.001**
At least once a week	1.87	1.08-3.25	0.03	**1.84**	**1.06-3.21**	**0.03**
Frequency of listening to radio						
Not at all	ref	ref		ref	ref	
Less than once a week	1.33	0.96-1.84	0.09	1.30	0.94-1.81	0.11
At least once a week	1.86	1.44-2.42	<0.001	**1.88**	**1.43-2.47**	**<0.001**
Frequency of watching TV						
Not at all	ref	ref		ref	ref	
Less than once a week	1.29	0.89-1.85	0.17	1.26	0.87-1.84	0.23
At least once a week	1.58	1.18-2.11	0.00	**1.55**	**1.14-2.09**	**0.01**
Had any STI’s in the last 12 months						
No	ref	ref		ref	ref	
Yes	1.02	0.71-1.45	0.93	1.00	0.70-1.45	0.97
Had genital sore/ulcer in the last 12 months						
No	ref	ref		ref	ref	
Yes	0.91	0.67-1.25	0.57	0.89	0.65-1.21	0.44
Had genital discharge in the last 12 months						
No	ref	ref		ref	ref	
Yes	0.85	0.66-1.09	0.20	0.87	0.67-1.13	0.30
Number of sex partners excluding spouse in last 12 months						
None	ref	ref		ref	ref	
1	0.94	0.71-1.25	0.67	1.06	0.80-1.42	0.67
2+	0.47	0.18-1.21	0.12	0.44	0.17-1.14	0.09
Condom used during last sex with most recent partner						
No	ref	ref		ref	ref	
Yes	1.54	0.95-2.49	0.08	1.60	1.00-2.56	0.05
Heard about other STI’s						
No	ref	ref		ref	ref	
Yes	1.64	1.20-2.24	0.00	**1.64**	**1.20-2.24**	**0.00**
Visited health facility last 12 months						
No	ref	ref		ref	ref	
Yes	1.48	1.19-1.84	<0.001	**1.47**	**1.18-1.84**	**0.00**
Visited by fieldworker in last 12 months						
No	ref	ref	0.00	ref	ref	
Yes	1.55	1.15-2.08		**1.50**	**1.12-2.02**	**0.01**
Heard of ART to treat HIV						
No	ref	ref		ref	ref	
Yes	4.04	2.82-5.78	<0.001	**3.86**	**2.69-5.54**	**<0.001**
Knowledge and use of HIV test kits						
Never heard of HIV test kits	ref	ref		ref	ref	
Has heard of HIV test kits	5.57	3.88-8.00	<0.001	**5.09**	**3.50-7.39**	**<0.001**
Knows HIV test kits but never tested with them	2.86	2.25-3.62	<0.001	**2.99**	**2.35-3.81**	**<0.001**

NB: Each exposure was fitted separately with the outcome while controlling for age, religion, ethnicity and working status

*COR *Crude odds ratio, *aOR* Adjusted odds ratio, *CI* Confidence interval

Further, women living in Northen compared to those in the Western Region were associated with greater odds of being aware of PrEP, (6.07 [2.94–12.55], *p* < 0.001). Cisgender women with higher education compared to those with no education were associated with 3.34 times greater odds of being aware of PrEP (3.34 [2.25–4.95], *p* < 0.001). Sexually active women living within the high-wealth quantile compared to those living in the low-wealth quantile were associated with 1.50 greater odds of being aware of PrEP (1.50 [1.17–1.93], *p* < 0.001). Relatedly, women who have read newspaper/magazine less than once a week (2.63 [1.75–3.95], *p* < 0.001) and at least once a week (1.84 [1.06–3.21], *p* = 0.03) were associated with greater odds of being aware of PrEP compared to those who have not. Also, Cisgender women who listened to the radio at least once a week had 1.88 times higher odds of being aware of PrEP compared to those who did not (1.88 [1.43–2.47], *p* < 0.001). Similarly, participants who watched television at least once a week had 1.55 times higher odds of being aware of PrEP compared to those who did not (1.55 [1.14–2.09], *p* = 0.01). Sexually active women who heard about other STIs were associated with greater odds of being aware of PrEP (1.64 [1.20–2.24], *P* < 0.001) compared to those who had not. Similarly, sexually active cisgender women who had visited a health facility (AOR = 1.47; 95% CI: 1.18–1.84; *p* < 0.001) or had been visited by a healthcare worker (AOR = 1.50; 95% CI: 1.12–2.02; *p* = 0.001) in the past 12 months had higher odds of being aware of PrEP. Women who had heard of ARTs to treat HIV were associated with greater odds of PrEP awareness (3.86 [2.69–5.54], *p* < 0.001), in the last 12 months. Results of our effect modification analysis (interaction between main exposure and potential effect modifiers) were not statistically significant (*p* > 0.05).

## Discussion

We investigated factors associated with awareness and attitude towards PrEP among sexually active cisgender women in Ghana using the 2022 Ghana Demographic and Health Survey (DHS) data. Our findings revealed low awareness and attitude towards PrEP (20.17%). Our finding is consistent with those reported in other studies in Nigeria (18.9%) [47, 48], Uganda (23.2%) [49], United States of America (USA) (21.0%) [50]. However, PrEP awareness among our sample was lower compared to those reported in Zambia (78.3%) [18], South Africa [51] and in the USA [52]. PrEP knowledge among our sample was however, higher than those reported in Malawi (15%) [39] and Tanzania (16.2%) [38] and across SSA (13.8%) [12].

The low level of PrEP awareness maybe partially due to limited education and inadequate awareness campaigns by health care providers [48, 53, 54].Additionally, difference in risk perception among participants across countries may explain the variation in PrEP awareness. These findings have important implications for the delivery of HIV prevention services in Ghana. There is an urgent need to strengthen interventions that promote awareness about PrEP and its proven effectiveness in preventing HIV transmission. This is particularly crucial given the evidence from clinical trials that demonstrated efficacy of both oral PrEP and the twice-yearly injectable PrEP (Lenacapavir) [55]. Peer-led targeted interventions should be scaled to increase awareness, uptake and adherence to PrEP particularly among those who are sexually active. In addition, culturally tailored and content specific messaging on PrEP should be developed, leveraging the reach of social media to drive awareness campaigns. Also, in-service training for clinicians should be implemented and prescribers encouraged to initiate PrEP conversations to increase awareness, uptake and adherence to PrEP.

We found that marital status was associated with PrEP awareness. Cisgender sexually active women who were married/living with their partners were associated with lower PrEP awareness. This aligns with findings from other studies (unadjusted models) that reported lower PrEP awareness among sexually active individuals [56–58]. One possible explanation is that married women or those living (or cohabitating) with their partners may have perceived themselves to be at lower risk of HIV, particularly when they believe that their partner is monogamous, and may therefore show less interest in learning about HIV prevention strategies such as PrEP. They may also view PrEP as primarily for individuals with multiple sexual partners or those perceived as being at risk, which could reduce their motivation to seek information about it. However, our finding contrasts with other studies that reported no association between marital status and PrEP awareness [12, 15, 38]. Conversely, in other studies, women engaged in sex work and living with their partners were found to be associated with higher odds of being aware of PrEP [36]. It is unclear what accounted for the lower odds of PrEP awareness in our study compared to some of the previous studies. It is plausible that HIV risk perception among the female sex workers particularly when cohabiting with their partners in those other studies compared to ours may have accounted for these different observations [36]. It is also plausible that female sex workers who perceived themselves to be at risk of HIV infection and sero-conversion were more likely than other study participants to seek information and become aware of HIV prevention strategies [36] compared to those in our sample. Given that married women and those living together in sub-Saharan Africa, including Ghana may be sexually active and face an elevated risk of HIV of exposure due to extra-relationship affairs and harmful gender norms [59–62], it is imperative to implement targeted HIV prevention strategies such as condom use and HIV self-testing among these groups.

We also found that women living in rural areas in Ghana were associated with lower odds of PrEP awareness. Our finding is consistent with reports of other studies [12, 63, 64]. Several factors may have accounted for this. For instance, women living in rural areas may face challenges associated with distance to the clinic, limited resources and transportation cost when accessing HIV preventive services [65, 66]. Similarly, women in rural areas experience stigmatizing situations which may discourage them from seeking HIV prevention services, including PrEP uptake [65]. Further, it is also plausible that healthcare providers in rural settings, may have limited awareness of PrEP due to inadequate training and may feel less empowered to initiate discussions about it [67]. Further, unavailability of informational and health promotion services in rural areas in Ghana may have accounted for the low level of PrEP awareness among sexually active women in our study. To increase awareness about PrEP among women in rural areas, efforts must be made to eliminate barriers that hinder access to HIV care. Similarly, interventions should aim to reduce inequalities that limit access to HIV prevention services, improve access to healthcare services, and address stigma faced by women living in rural areas [64]. This is critical given that socially and economically empowered women are more likely to negotiate sexual decisions with their partners, an important strategy along the HIV prevention and care continuum [68, 69].

We also found that women in Volta and Northern regions were highly aware of PrEP compared to those in the Western region. Our finding is supported by results from Burkina Faso [15]. One plausible explanation is the presence of health infrastructure supported by health-related non-governmental and charity organizations in these regions, which may have influenced PrEP information services. This finding also highlights the importance of considering regional variations when designing and implementing of HIV prevention interventions.

Women with higher level of education had greater odds of being aware of PrEP compared to those with no education. This aligns with previous studies showing that women with higher education are more likely to be aware of PrEP [15, 64]. Educated women may have better access to health information and higher health literacy which can enhance their knowledge of PrEP [12]. Similarly, higher educational attainment is often linked to improved access to healthcare services and increased utilization [70]. Together, greater access to health information and higher literacy may contribute to increased awareness of PrEP and HIV prevention services among women in Ghana. To ensure no one is left behind, PrEP awareness interventions must intentionally target women with lower educational attainment. These efforts could be implemented through the Community-Based Health Planning Services (CHPS) framework, where community health workers and volunteers deliver PrEP education in local languages within community settings [71].

Our findings also showed that women with higher income reported greater PrEP awareness. This aligns with other reports reporting that participants in the richer and richest wealth quantiles had higher odds of PrEP and HIV awareness compared to those in the poorest quantiles [12, 72, 73]. Conversely, a study in Tanzania found no association between wealth index and PrEP awareness or uptake [74], possibly reflecting cultural or contextual differences. Among our participants, women from wealthier households may be better positioned socioeconomically and have better access to healthcare services, including HIV prevention [12]. This suggest that socioeconomic status influences access to and uptake of health promotion and illness prevention services including PrEP. Health managers should therefore implement policies to reduce inequalities and disparities in access to healthcare and HIV prevention services. Such policies must prioritize and target vulnerable populations in lower income brackets in Ghana to increase equitable access to PrEP care.

Our analysis also showed that women with access to media (newspaper, radio and television) had higher odds of being aware of PrEP compared to those without media access. This finding is consistent with reports from other studies [13, 15, 75, 76] and highlights the critical role that both traditional and social media can play in dissemination health information, including HIV prevention strategies. Policymakers including the ministry of health (MOH) and other relevant agencies should adopt targeted media campaigns to raise awareness about PrEP. Leveraging mainstream and social media such as Facebook, Twitter, TikTok, community radio and information systems could significantly enhance knowledge of HIV prevention including, PrEP uptake and continuation [76–78]. Such an approach could complement existing strategies aimed at increasing PrEP awareness and uptake.

We also found that women who had heard about other STIs were more likely to be aware of PrEP, consistent with previous studies [12, 79]. Similarly, women who visited health facility or were visited by healthcare workers had higher odds of PrEP awareness. This may be because the women were more likely to be exposed to HIV prevention and PrEP information during these encounters. These findings underscore the importance of integrating PrEP discussions into routine STI screening and healthcare visits. We recommend that policymakers promote healthcare worker outreach especially in remote and underserved communities, to raise awareness about PrEP. Additionally, strengthening, resourcing and enhancing existing Community-Based Health Planning Services (CHPS) service delivery models could help extend PrEP and HIV prevention services to those in rural areas.

### Strengths and limitations of our study

Our study has some strengths. A key strength is the use of a large, population level dataset, which enhances the generalizability of our findings to women in Ghana. We also fitted separate regression models for each exposure-outcome relationship while adjusting for potential covariates. This approach allowed us to estimate the independent effect of each exposure without the risk of confounding or interaction effects that may arise when multiple exposures are included in the same model [42, 43], thereby strengthening the validity of our findings.

However, our study has some limitations. The cross-sectional design limits our ability to establish causality. In addition, the use of self-reported data may have introduced recall and social desirability biases [80, 81]. Finally, unavailability of data on actual PrEP use within the 2022 GDHS dataset limited our ability to assess whether awareness translated into uptake.

## Conclusions

Our analysis found that PrEP awareness among sexually active cisgender women in Ghana was generally low. Factors such as marital status, geographical location, educational attainment, wealth index and access to media were independently associated with both awareness of and positive attitudes towards PrEP. Further, awareness of STIs, visiting healthcare facility, receiving visits from field workers, awareness about anti-retroviral therapies (ARTs) and HIV test kits were associated greater with PrEP awareness among sexually active cisgender women in Ghana. We also found that condom use with the most recent sexual partner was low among sexually active women. These findings underscore the need for targeted interventions that address these determinants of PrEP awareness. Policymakers and stakeholders should design and implement health literacy campaigns using community outreach, media advertisements, and peer education strategies to increase PrEP awareness, particularly among sexually active cisgender women in Ghana.

## Data Availability

The datasets supporting the conclusions of this article is available in the demographic and health survey (DHS) repository which is publicly available at [https://dhsprogram.com](https:/dhsprogram.com).

## Abbreviations

PrEP: Pre-exposure prophylaxis
GDHS: Ghana demographic and health survey
PHC: Population and housing census
STI: Sexually transmitted infections
ART: Anti-retroviral therapy
CHPS: Community-based health planning services
EAs: Enumeration areas
